# Safety profile of inactivated COVID-19 vaccine in indonesian adults

**DOI:** 10.1016/j.jvacx.2023.100331

**Published:** 2023-06-10

**Authors:** Hindra Irawan Satari, Nastiti Kaswandani, Bernie Endyarni Medise, Julitasari Sundoro, Sri Rezeki Hadinegoro, Elcha Leonard, Ade Putra, Putra Fajar Angkasa

**Affiliations:** a National Committee of Adverse Event Following Immunization (NC-AEFI) Indonesia, Central Jakarta, Indonesia; b Department of Pediatrics, Faculty of Medicine, University of Indonesia, Central Jakarta, Indonesia

**Keywords:** Inactivated vaccine, AEFI, COVID-19

## Abstract

**Background:**

Vaccines are urgently needed to handle the morbidity and mortality of the COVID-19 pandemic in Indonesia. The inactivated vaccine is widely used in Indonesia's national immunization program due to its eligibility of stock, easier to transport, and considered to be more established than newer platforms. In this study, we aimed to evaluate the safety profile of the inactivated vaccine and analyze the safety profile between adults and the elderly.

**Methods:**

A prospective analytical study was conducted to evaluate the safety profile of inactivated COVID-19 vaccine among healthy adults aged ≥ 18 years from September 2nd to December 28th, 2021, at ten primary health centers from 5 districts in Jakarta, Indonesia. The participants were instructed to record the symptoms after inactivated COVID-19 vaccine injection in the diary card for 28 days. Chi-square tests were carried out to analyze the relationship between the adverse event following immunization (AEFI) in adults and elderly groups.

**Results:**

Four of 1113 participants were not included in this study due to the lack of follow-up. Out of 1109 participants, there were 1044 adults (18–59 years) and 65 elderly (>59 years). There were no serious AEFI cases reported. Most AEFI cases were mild to moderate and resolved after several days of injection. Local pain, myalgia and fatigue were the most frequent adverse events reported. We found that there was no correlation between the adults and elderly age group with the incidence of AEFI (p = 0.924) for local reactions (p = 0.181) and most of the systemic reactions (p = 0.629). However, there is an increased risk of fever in the elderly group compared to the adult group (OR 4.046, 95 % CI 1.794–9.124, p = 0.003) following immunization.

**Conclusions:**

Our study demonstrated that the inactivated COVID-19 vaccine is safe, considering that all symptoms experienced were mild to moderate and resolved entirely.

## Highlights

• There were no serious AEFIs reported after 28 days of observation.
• Most of the adverse reactions were mild to moderate in severity.
• Further research is needed to analyze the cause of the elevated risk factors in the elderly compared to other groups,

## Introduction

In response to the SARS-CoV-2 outbreak infection in Wuhan, China, which led to the COVID-19 pandemic, prevention measures, including vaccination, are needed to decrease the morbidity and mortality of COVID-19 [1], [2]. In parallel with the imposed restrictions to prevent viral spread by using masks and social distancing, the vaccine was developed to avoid potential viral damage and initiate the immunity system against the SARS-CoV-2 virus. Several vaccine platforms have been developed for COVID-19, such as inactivated and recombinant proteins, including newer vaccine platforms, viral vectors, and mRNA vaccine [1], [2].

Sinovac Biotech's (inactivated vaccine, CoronaVac®) has been approved as one of the vaccines for use in Indonesia's national COVID-19 immunization program according to the Emergency Use Authorization (EUA) by the National Regulatory Authority on January 11th, 2021 [3]. The inactivated vaccine is the most common and mature way of developing new vaccines. Therefore, it is considered to have more established and better-documented safety profiles, including adverse events following immunization, than any other vaccine platform [2], [4], [5], [6], [7]. CoronaVac® is also widely used in Indonesia due to its eligibility of stock and easier to transport (cold chain) to all of Indonesia's region [8].

Current evidence about the safety of inactivated COVID-19 vaccines from phase I - III randomized controlled trials in several countries showed that the vaccine is safe to use [9], [10], [11], [12], [13], [14]. The Phase I/II clinical trial in China reported that the most common local and systemic adverse reactions were pain at the injection site and fever [9]. All of the symptoms reported in Phase I–II are mild and not last longer than several days. Meanwhile, in phase III in Indonesia, the most frequent AE (Adverse Event) was local pain at the injection site [14], [15].

A vaccination program's effectiveness depends on the vaccination dose given. Therefore the willingness to be vaccinated in the population is very important. Vaccine acceptance and desire in the general population are associated with several factors, such as the perceived risk of infection, concern regarding its side effects, personal beliefs, religious reasons, sociocultural dynamics, and socio-economic status [16], [17].

As of September 1st, 2021, COVID-19 Handling and National Economic Recovery Committee reported 27.58 % of the elderly (>60 years old) had received the COVID-19 vaccine in the first dose and 19.30 % for the second dose nationwide [18]. In Jakarta, 93.34 % of the elderly have been vaccinated with the first dose and 78.93 % with the second dose [18]. Although the vaccination rate in Jakarta was quite close to the target, the national vaccine rate for the elderly was far from expected, which is 70 % of the total population, to end the acute phase of the pandemic. World Health Organization recommends high vaccination coverage rates should be achieved for all older adults [19].

In this Post Authorization Safety Study (PASS), we aimed to evaluate the vaccine's safety and benefit-risk profile and support regulatory decision-making after the vaccine has been authorized and given to more than one million dose injections in Indonesia. We also aspired to analyze and compare the vaccine safety data between adults and elderly age groups to find the correlation between age and the symptoms after the inactivated COVID-19 vaccine injection.

## Method

### Study design, period and setting

A prospective analytical study evaluated the safety profile of inactivated COVID-19 vaccines among healthy adults aged ≥ 18 years from September 2nd, 2021, to December 28th, 2021, at ten primary health centers from 5 districts in Jakarta, Indonesia. The provincial health office appointed ten primary health centers as the study sites based on the largest proportion of Sinovac/Coronavac used in those primary health cares during the study period.

We analyzed the number and percentage of subjects with each local reaction (local pain, redness, induration, swelling, others) and systemic events (fever, myalgia, fatigue, others) with three categories (mild, moderate, severe) within three days and 28 days after vaccination. Each symptom's mild, moderate, and severe category was classified using the Brighton Collaboration Case Definition in Table 1 below [20], [21]. We also analyzed serious AEFIs in subjects that occurred at the time of observation.

**Table 1:** Case Definitions for Adverse Events Following Immunization (AEF_).

Symptoms	Mild	Moderate	Severe
Local Pain	Mild pain to touch	Pain with movements	Significant pain at rest
Redness/swelling/induration	Reaction wholly included in the small circle	The largest diameter of the reaction included between the two circles	Reaction beyond the largest circle
Fever	38.0–38.4 C	38.5–38.9 C	≥39.0 C
Fatigue Myalgia	No interference with activity	Some interference with activity	Significant prevents daily activity
Other systemic events*	No interference with activity	Some interference with activity but not requiring medical intervention	Prevents daily activity, requires medical intervention

*Other systemic events should refer to the published case definition in Brighton Collaboration Definition.

### Source population, inclusion and exclusion criteria

The source population for the study was all adults aged ≥ 18 years who received CoronaVac® injections during the study period in 10 selected primary health centers in Jakarta, Indonesia. People were excluded from the study if: concomitantly enrolled or scheduled to be enrolled in another trial; evolving mild, moderate, or severe illness, especially infectious disease or fever (body temperature ≥ 37.5℃, measured with thermometer); women who are lactating, pregnant or planning to become pregnant during the study period; had a history of uncontrolled coagulopathy or blood disorders contraindicating intramuscular injection; have any account of confirmed or suspected immunosuppressive or immunodeficient state, or received in the previous four weeks a treatment likely to alter the immune response (intravenous immunoglobulins, blood-derived products or long-term corticosteroid therapy (>two weeks); receive any vaccination within one month before and after the investigational product (IP) immunization; or plan to move from the study area before the end of the study period.

### Data collection

Data were collected using a self-administered method. On the day of vaccination, all subjects were given diary cards to assess and record information for the appearance, duration and intensity of any local and systemic reaction event expected for 28 days following the immunization, with particular attention within the first three days. The local reaction and systemic event assessment involved daily axillary temperature readings in the first three days and the measurement of local response if it occurred. Each subject was also supplied with a thermometer and plastic bangle and then instructed on how to use it. Safety data from the diary card is monitored by a field investigator within three days after the immunization via telephone.

After 28 days of observation, all of the diary cards were collected from the participants. The participants were asked to collect the diary cards to be exchanged with the goody bags as the appreciation gift for the participants after completing the diary cards.

### Statistical analysis

The collected data were coded, entered into MsExcel, and exported to IBM SPSS statistics 22.0 software (SPSS Inc., Chicago, IL, USA) for data cleaning and analysis. Descriptive analysis was calculated, such as frequency distribution and proportions of subject characteristics, local and systemic reactions, and serious adverse events. We used the chi-square test to assess the relationship between the adverse event following immunization in adults and elderly groups. We considered a *p*-value of < 0.05 supposed to represent a statistically significant and reported 95 % Confidence interval (CI) for odds ratio (OR).

### Ethical approval

This study has been reviewed and approved by The Health Research Ethics Committee of the Faculty of Medicine of the University of Indonesia (Reference KET-361/UN2.F1/ETIK/PPM.00.02/2021).

## Results

### Study subjects

**Fig. 1:**
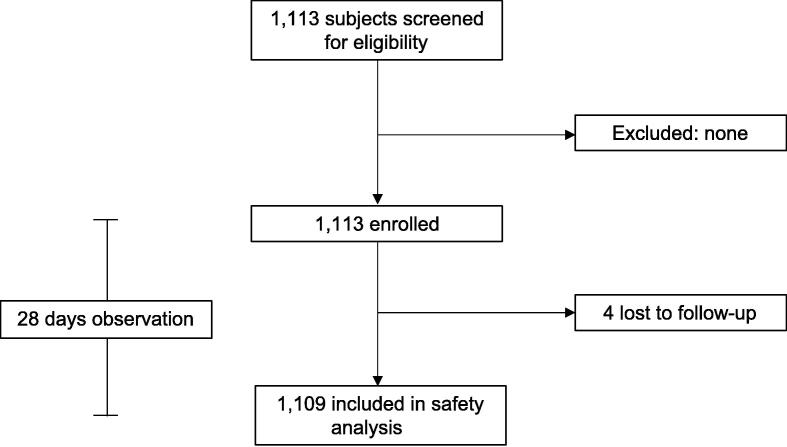
The Data Collection Process.

A total of 1113 subjects were enrolled in this study. However, due to the lack of follow-up, the analysis did not include four subjects. The data collection process showed in the Fig. 1. The final sample of 1109 subjects enrolled in the study consisted of 534 males and 575 females. The main characteristics of the participants are shown in Table 2 below. The mean of the subject's age and standard deviation were 36.92 ± 13.26 years. Most subjects were in the age group 18–30 years (37. 5 %).

**Table 2:** Demographic Characteristics.

Description	Dose-1	Dose-2	Total
N included	3	1106	1109
Gender			
Male	1 (33.33 %)	533 (48.19 %)	534 (48.15 %)
Female	2 (66.67 %)	573 (51.81 %)	575 (51.85 %)
Age (years)			
Mean ± SD	44.81 ± 20.40	36.90 ± 13.23	36.92 ± 13.26
Min; max	25; 73	18; 90	18; 90
Age group (years)			
18–30	1 (33.33 %)	415 (37.52 %)	416 (37.51 %)
31–45	1 (33.33 %)	376 (34.00)	377 (34.00 %)
46–59	0	251 (22.69 %)	251 (22.63 %)
>59	1 (33.33 %)	64 (5.79 %)	65 (5.86 %)

### Safety

**Graph 1:**
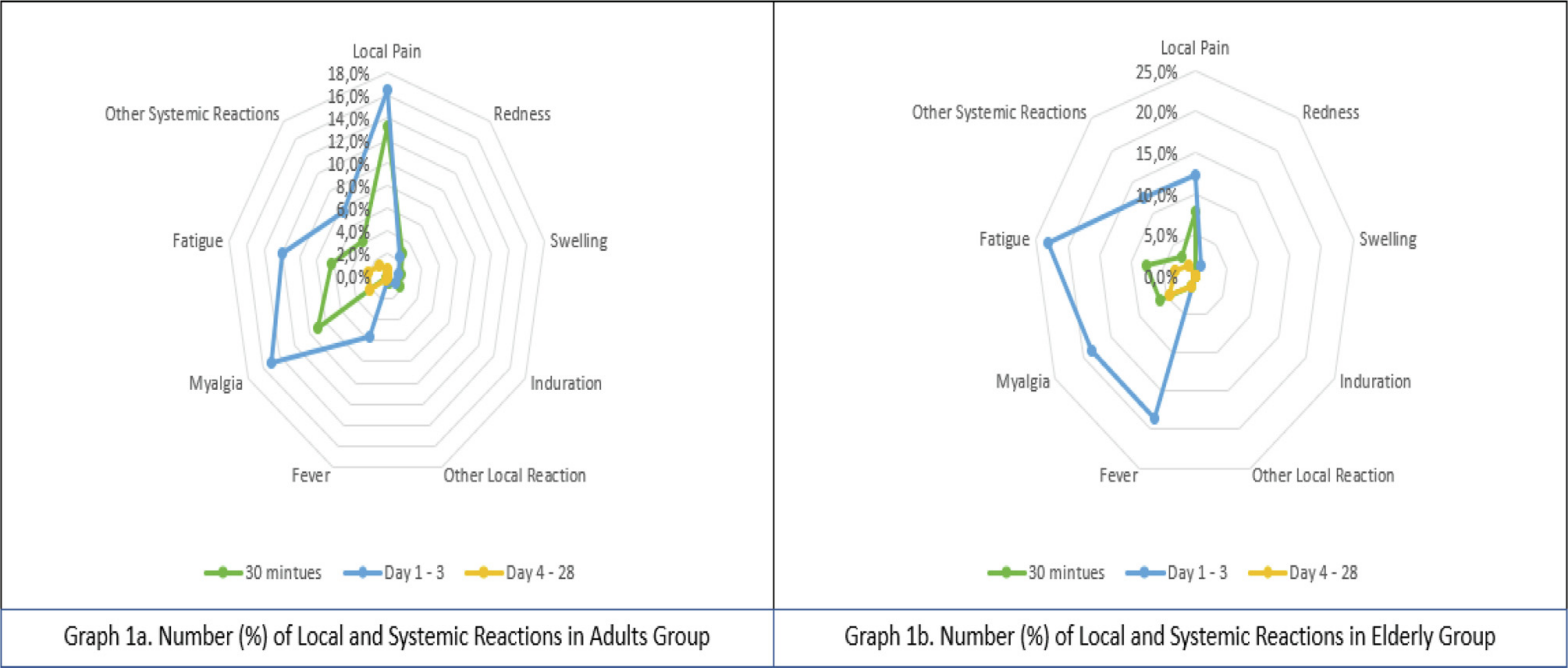
Number (%) of Events with Local and Systemic Reactions Following Vaccination.

During 28 days of observation, 188 subjects experienced local solicited reactions, and 163 subjects with systemic solicited reactions. (Table 3) The total number of subjects who experienced at least one local reaction was 17.7 % (n = 196), and any systemic reaction was 17.0 % (n = 189). The most frequently reported local reaction events were local pain in the mild category on days 1–3 (7.1 %), and the systemic reaction was myalgia in the mild category on days 1–3 (12.0 %). There was a higher percentage of local reactions in adults than in the elderly group. (Graph 1).

**Table 3:** Solicited and Unsolicited Reactions in Total Age Group.

Reaction	Local		Systemic	
	Yes	No	Yes	No
Solicited	188	856	163	881
Unsolicited	8	1,036	59	985

The other local reactions reported were itch (0.5 %), bleeding at the site of injection (0.2 %), and bruises (0.1 %). The total of other systemic reactions events experienced by the participants consists of vertigo (2 %), drowsiness (1.3 %), headache (0.7 %), nausea (0,5 %), an increase of appetite (0.3 %), arthralgia (0.3 %), lethargic (0.2 %), diarrhea (0.2 %), and itch (0.2 %).

In the elderly group, out of 65 people, the total number of subjects who experienced at least one local reaction was 10.8 % (n = 7), and any systemic event was 20.0 % (n = 13). Overall, the most frequently reported local reaction events were local pain in the mild category on days 1–3 (9.2 %), and the systemic reaction was fatigue in the mild category (18.5 %).

Analyses of adverse events were done in the adults (18–59 years) and elderly (>59 years) age groups. We found no difference in risk between the adults and elderly age groups with the incidence of AEFI (p = 0.924) for local and most systemic reactions. However, there is an increased risk of fever in the elderly group compared to the adult group (OR 4.046, 95 % CI 1.794–9.124, p = 0.003) following immunization (Table 4). Both groups showed that the fever gradually decreased after several days. In adults, the fever resolved on day 6. Meanwhile, the fever entirely resolved on day 5 in the elderly group.

**Table 4:** Correlation of AEFIs incidence between adults and elderly group.

A			
Variable	Adverse Event		OR (95 % CI) p-value
Age Group	None (%)	Yes (%)	
18–59	785 (75.2)	259 (24.8)	1.073 (0.607–1.899) 0.924
>59	48 (73.8)	17 (26.2)	

**Variable**	**Local Reaction**		**OR (95 % CI)** **p-value**
**Age Group**	**None** **(%)**	**Yes** **(%)**	

18–59	855 (81.9)	189 (18.1)	0.546 (0.245–1.215) 0.181
>59	58 (89.2)	7 (10.8)	

**Variable**	**Systemic Reaction**		**OR (95 % CI)** **p-value**
**Age Group**	**None** **(%)**	**Yes** **(%)**	

18–59	898 (83.1)	176 (16.9)	1.233 (0.657–2.313) 0.629
>59	52 (80.0)	13 (20.0)	

B			
Variable	Fever		OR (95 % CI) p-value
Age Group	None (%)	Yes (%)	
18–59	1,009 (96.6)	35 (3.4)	4.046 (1.794–9.124) 0.003
>59	57 (87.7)	8 (12.3)	

**Variable**	**Fatigue**		**OR (95 % CI)** **p-value**
**Age Group**	**None** **(%)**	**Yes** **(%)**	

18–59	957 (91.7)	87 (8.3)	1.119 (0.470–2.665) 0.982
>59	59 (90.8)	6 (9.2)	

**Variable**	**Myalgia**		**OR (95 % CI)** **p-value**
**Age Group**	**None** **(%)**	**Yes** **(%)**	

18–59	931 (89.2)	113 (10.8)	0.838 (0.354–1.984) 0.845
>59	59 (90.8)	6 (9.2)	

**Variable**	**Other Systemic Reaction**		**OR (95 % CI)** **p-value**
**Age Group**	**None** **(%)**	**Yes** **(%)**	

18–59	985 (94.3)	59 (5.7)	0.808 (0.246–2.650) 1.000
>59	62 (95.4)	3 (4.6)	

C			
Variable	Local Pain		OR (95 % CI) p-value
**Age Group**	**None** **(%)**	**Yes** **(%)**	
18–59	865 (82.0)	179 (17.1)	0.583 (0.262–1.299) 0.244
>59	58 (89.2)	7 (10.8)	

**Variable**	**Redness**		**OR (95 % CI)** **p-value**
**Age Group**	**None** **(%)**	**Yes** **(%)**	

18–59	1,013 (95.0)	31 (3.0)	0.511 (0.069–3.800) 1.000
>59	64 (98.5)	1 (1.5)	

**Variable**	**Induration**		**OR (95 % CI)** **p-value**
**Age Group**	**None** **(%)**	**Yes** **(%)**	

18–59	1027 (98.4)	17 (1.6)	0.448 (0.026–7.546) 0.618
>59	65 (100.0)	0 (0.0)	

**Variable**	**Swelling**		**OR (95 % CI)** **p-value**
**Age Group**	**None** **(%)**	**Yes** **(%)**	

18–59	1026 (98.3)	18 (1.7)	1.438 (0.025–7.107) 0.619
>59	65 (100.0)	0 (0.0)	

**Variable**	**Other Local Reaction**		**OR (95 % CI)** **p-value**
**Age Group**	**None** **(%)**	**Yes** **(%)**	

18–59	1036 (99.2)	8 (0.8)	0.930 (0.053–16.304) 1.000
>59	65 (100.0)	0 (0.0)	

**Fig. 2:**
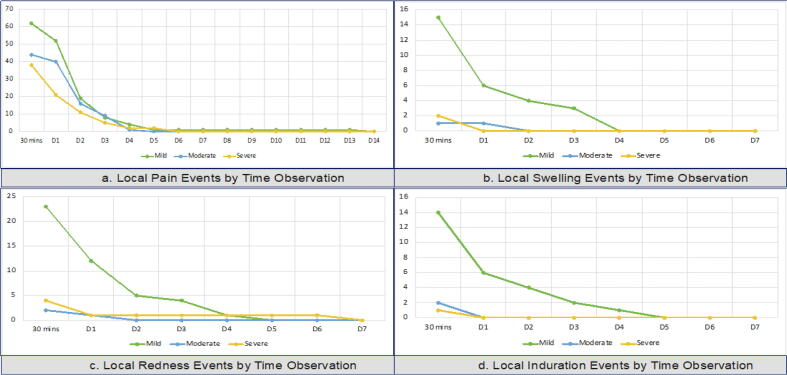
The time observation of local reaction events. The graph showed the real observed time of the local reaction occurrence in all of the participants.

The local reactions that occurred during the observation time are presented in Fig. 2. Most of the local reaction events reported were in the mild category at 30 min of observation. Most of the symptoms were resolved and disappeared in several days. No local swelling and induration events were reported to subjects above 59 years. Most reported other local reaction events (such as itchy, sore, bruises, and bleeding) were in the mild category at 30 min of observation (0.3 %).

**Fig. 3:**
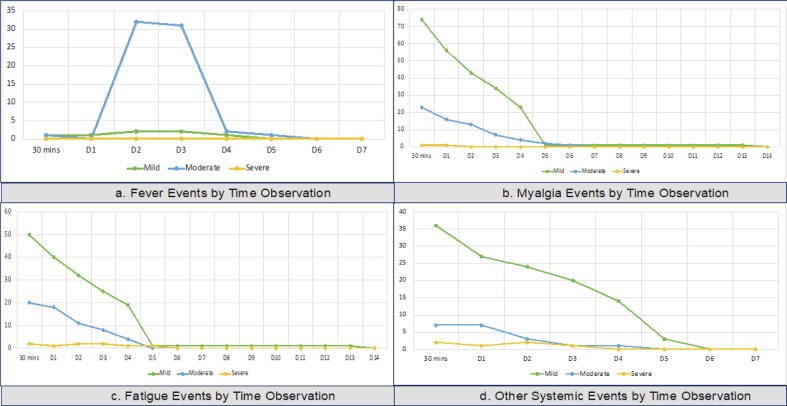
The time observation of systemic reaction events. The graph showed the real observed time of the systemic reaction occurring in all of the participants.

Fig. 3 shows the systemic reactions that occurred by the time observation. The most frequently reported event is myalgia in the mild category at 30 min of observation (6.9 %). It decreased from day one and disappeared on day 14. Other systemic events such as headache, dizziness, arthralgia, diarrhea, nausea, drowsiness, lethargy, itch, increased appetite, and stiffness were mainly reported in the mild category at 30 min of observation (3.25 %). It decreased significantly from day one and disappeared on day 6.

## Discussion

This study is an active AEFI surveillance after more than one million dose injection of inactivated COVID-19 vaccine in Indonesia. It is essential to ensure the safety profile of CoronaVac because it's the majority of inactivated COVID-19 vaccines to be used in Indonesia. In this study, four subjects who received inactivated COVID-19 vaccine in the first and 1,105 subjects in the second dose were observed in 28 days. There were more subjects with the second dose than with the first dose because the study's observation period matched the second dose's vaccination schedule in Jakarta. We observed and followed up with each subject daily through the diary cards so the onset and resolved time of every local and systemic reaction were well documented. Additionally, there were participants from different risk groups, including the groups of elderly (>59 years old), displaying the study results more generalizable to the real-world context.

There were no serious AEFI (SAE) cases reported. We found that the most commonly reported local reactions for adult and > 59 years old groups were local pain and the entire subjects group. Meanwhile, the most frequently reported systemic reactions are fatigue for the elderly and myalgia for all the subjects.

A lower percentage of adverse events were reported in the elderly group in the local reactions compared to those in the younger and productive age group. It is aligned with the theory that the elderly group would have fewer adverse events considering the lack of immune cell responses to the vaccine [22]. It is also similar to the meta-analysis results showing a lower incidence of local and systemic AEs in older people than in the young, suggesting that COVID-19 vaccines are practical and have more advantages in the safety index for the elderly [23]. However, our analysis showed no difference in risk between adults and the elderly experiencing local reactions following immunization.

On the other hand, we have found an increased risk of having a fever in the elderly group compared to the adult group following immunization. Compared to another study in United Arab Emirates (UAE) reported that there is no difference in risk of having fever in the age group (≤49 and >49 years old) after the immunization of other COVID-19 inactivated vaccines (Sinopharm) [24]. The difference might be due to the smaller population in the elderly group. Further observation and analysis might be needed in the larger population to support the data.

Our results were similar to data published in Chile, Brazil, Thailand, Turkey, and China about inactivated vaccines. Most of the adverse events were mild to moderate in severity and resolved after several days [6], [12], [25], [26], [27], [28], [29]. We found that the most common AE was local pain at the injection site, mostly of mild severity, which generally commenced after the injection and resolved within a few days. The most frequently reported systemic adverse event is myalgia. There was no case of anaphylaxis occurred. The symptoms resolved within several days, and no SAEs were reported.

Supangat et al. have conducted research about AEFI of Coronavac in Jember, one of the cities in Indonesia. This study showed similar results to ours in that the AEFI of CoronaVac was mainly in the mild category, and the most common AEFI of SARS-CoV-2 vaccinations was localized pain in the injection site during the first and booster doses [30]. This study involved a smaller sample size with specific populations (medical clerkship students) than ours. As the study subjects in Supangat's study were medical clerkship students, they would provide more precise and detailed symptoms compared to non-medical-knowledged people [30]. Although our studies have different characteristics on the subject's educational background, the similarity of the results showed the validity of our research as the AE reported is relatively the same. Furthermore, the heterogeneous population in our study would provide a better approach to rendering to the real-world context.

There were uncommon findings for other systemic reactions: drowsiness and increased appetite. There were 14 events of drowsiness and three reported events of increased appetite. The participants experienced the symptoms 30 min and 1–3 days after the injection. The symptoms resolved after several days. Our findings were similar to Benjamanukul et al., who also reported hunger and hypersomnia in participants after immunization [6]. Simatupang et al. also reported other symptoms like headache, fever, shivering, sleepiness, nausea, dysphagia, and cold as the adverse event following immunization, similar to our results [31]. The following results might indicate that drowsiness and increased appetite should be considered adverse events after COVID-19 inactivated vaccine immunization without serious risk.

Differing from other vaccine platforms, inactivated vaccine seems to have the lowest rate of reported AE compared to other platforms such as vector vaccines and mRNA vaccines [2], [7]. Moreover, when comparing vaccine type and AEFI, other vaccines, such as Corminaty to CoronaVac, there was associated with 83 %-reduced odds of any adverse reactions in CoronaVac [AOR = 0.17, 95 % CI 0.15–0.20], 92 %-reduced odds of local adverse reactions (AOR = 0.08, 95 % CI 0.06–0.09), and 76 %-reduced odds of systemic adverse reactions (AOR = 0.24, 95 % CI 0.16–0.28) [2], [7].

The strength of our research is the active surveillance of adverse events after immunization by daily monitoring and close observation reported in the daily card. Therefore it is possible to capture mild and moderate events after the immunization. Although we had thoroughly analyzed our data to draw a fair interpretation, our study had a few limitations. The location of the study is only in one province in Indonesia. The method to evaluate the adverse events in every participant is using a diary card, so there might be subjectivity in filling out the diary card. The analysis compared the risk of adults and elderly age groups following immunization. However, there were a distinctive number of subjects in the adult and elderly age group. In contrast, out of 1109 subjects, the total number of elderly participants in this study was 65, which might confound the analysis.

## Conclusion

In summary, most AEFI cases were mild to moderate and resolved after several days of injection without any symptoms left. Local pain, myalgia, and fatigue are the most frequent adverse events reported in this study. There were no SAEs reported. A lower percentage of adverse events was reported in the elderly group in the local reactions compared to those in the younger and productive age group. However, there was no difference in risk between the adults and elderly age groups with the incidence of AEFI for local and most systemic reactions. Although, we found an increased risk of fever in the elderly group compared to the adult group following immunization. Further studies with larger subjects were still needed to examine whether fever is related to the age group as the adverse events following immunization that occurred after the immunization. Our study concluded that the inactivated COVID-19 vaccine is safe to use.

## Funding source

This study was a grant from PT BioFarma with number 008.26/EGD/IV/2021, PO/BIOF/2021/1289, PO-00032090, April 26th 2021. The sponsor had no role in the study design, collection, analysis, or interpretation of data, writing of the report, or decision to submit the article for publication.

## Declaration of Competing Interest

This study was a grant from PT BioFarma. However, sponsor had no role in the study design, collection, analysis or intepretation of the data, writing of the report or decision to submit the article for publication. The authors have no known competing financial interest that could have appeared to influence the study result reported in this paper

## Data Availability

Data will be made available on request.
