# Supplementary figures and images for: Safety profile of inactivated COVID-19 vaccine in indonesian adults
Source: Vaccine X. 2023 Jun 10;14:100331. doi: 10.1016/j.jvacx.2023.100331 (PMC10257516; doi:10.1016/j.jvacx.2023.100331)

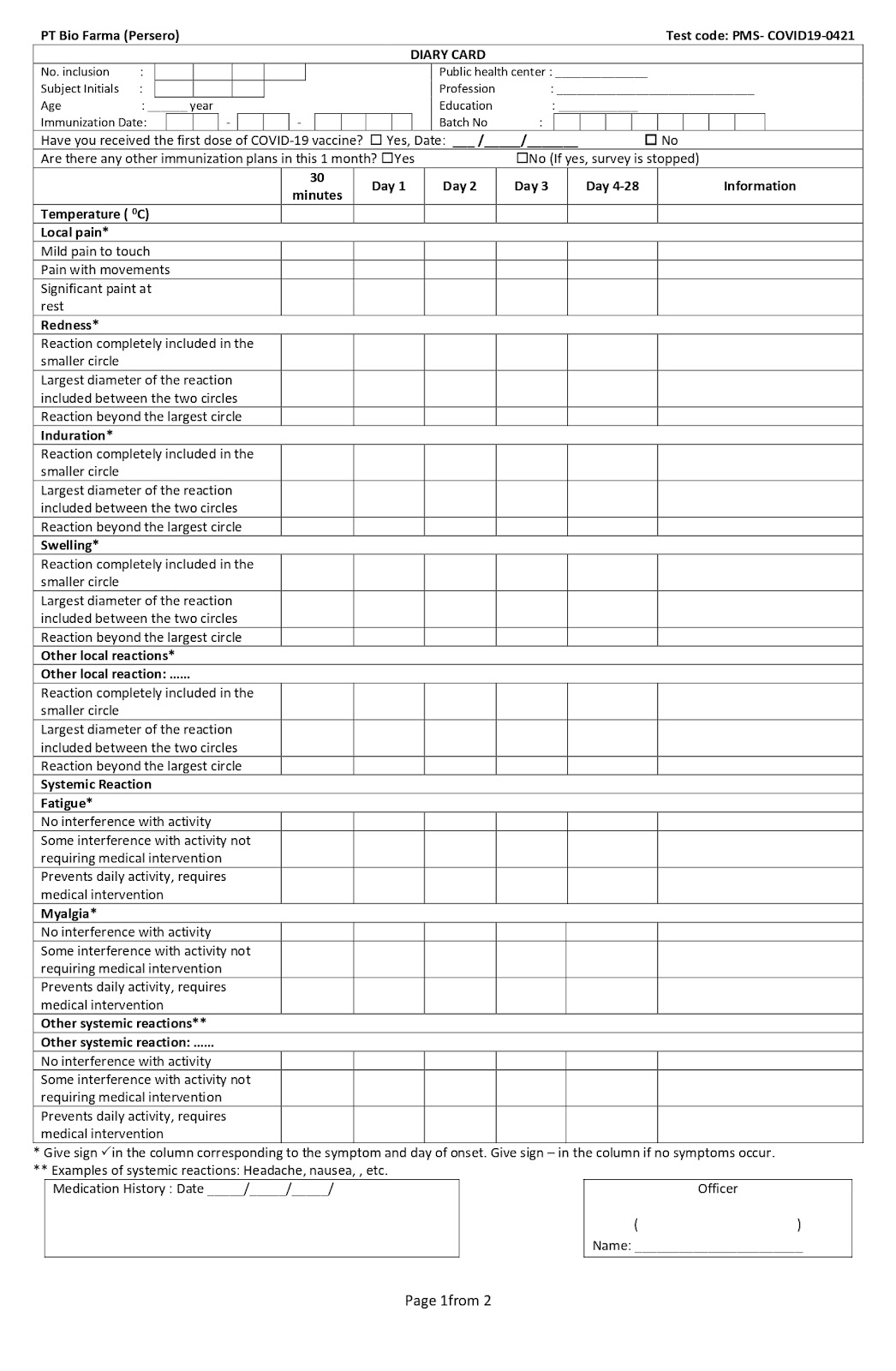


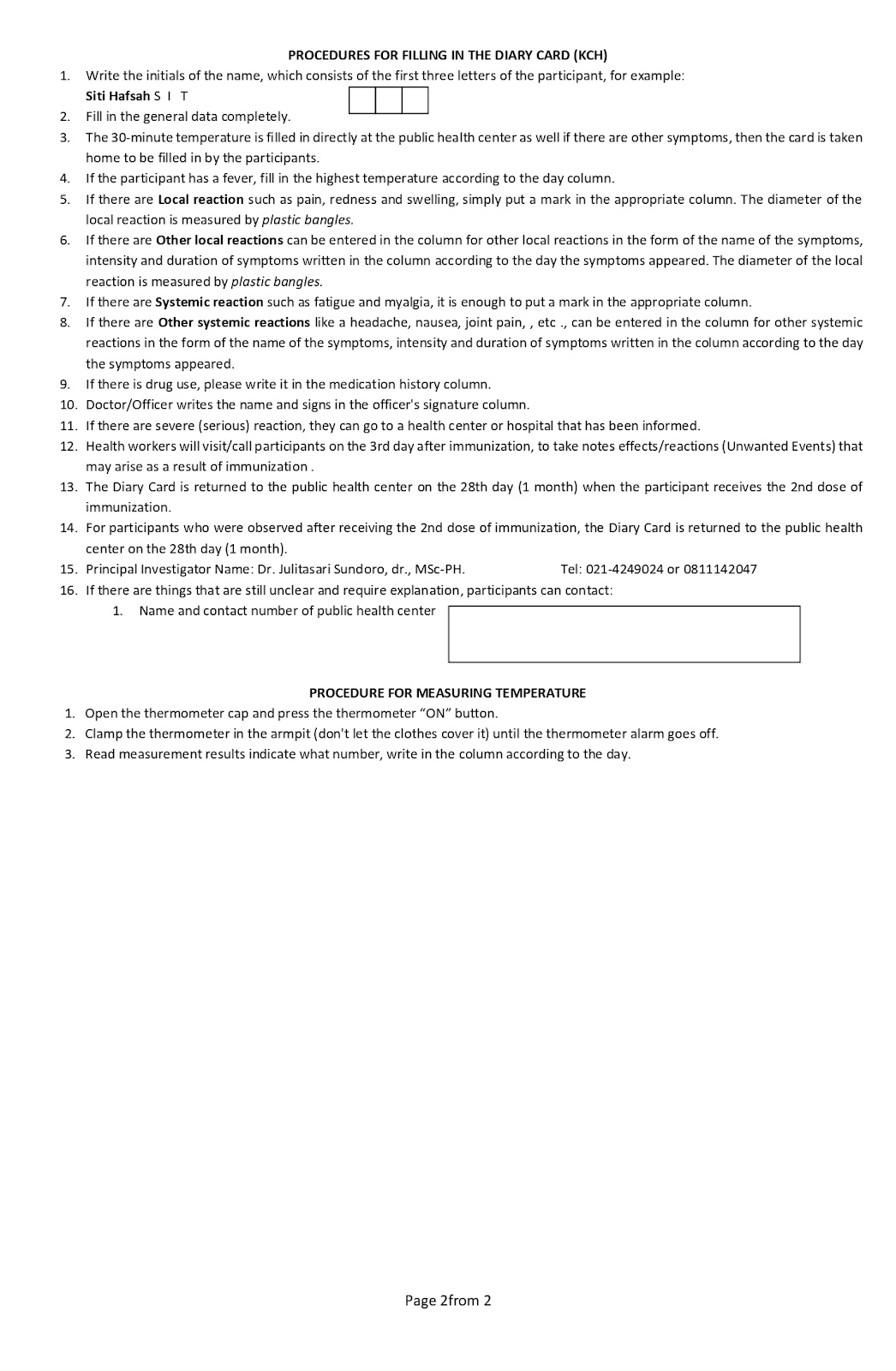

Supplement: Supplementary data 1 [file mmc1.docx]
